# Psychoeducational Program Increases Telomerase Activity in Bipolar Disorder: A Gender‐Based Randomized Controlled Trial

**DOI:** 10.1111/cns.70292

**Published:** 2025-04-09

**Authors:** G. Massa, F. Bruno, L. Tarsitani, M. Caredda, M. Biondi, A. Bevilacqua, S. Canterini

**Affiliations:** ^1^ Division of Neuroscience, Department of Psychology University La Sapienza Rome Italy; ^2^ PhD Program in Behavioral Neuroscience Sapienza University of Rome Rome Italy; ^3^ Deparment of Human and Social Sciences, Faculty of Social and Communication Sciences Universitas Mercatorum Rome Italy; ^4^ Department of Human Neurosciences, Faculty of Medicine and Dentistry Sapienza University of Rome Rome Italy; ^5^ Department of Dynamic, Clinical Psychology and Health Studies Sapienza University of Rome Rome Italy; ^6^ Systems Biology Group Lab and the Experts Group on Inositols in Basic and Clinical Research (EGOI) Research Center in Neurobiology Daniel Bovet (CRiN) Rome Italy; ^7^ European Center for Brain Research IRCCS Fondazione Santa Lucia Rome Italy

**Keywords:** bipolar disorder, cellular aging, chronic stress, gender differences, intervention efficacy, mental health, psychoeducation, randomized controlled trial, telomerase activity, telomere shortening

## Abstract

**Aims:**

This randomized controlled trial evaluated the efficacy of a psychoeducational program in enhancing telomerase activity (TA) among patients with bipolar disorder (BD), with a specific focus on gender differences.

**Methods:**

A total of 62 participants were assigned to either the psychoeducation (PE) group or the control (CTR) group, with TA measured both before and after the intervention.

**Results:**

Results demonstrated a significant increase in TA in the PE group compared to the controls at the conclusion of the study. Notably, gender‐specific analyses revealed that female participants showed significant increases in both TA and delta TA (Δ_TA_), with Δ_TA_ PE = 0.586 ± 0.273 and Δ_TA_ CTR = −0.251 ± 0.177. In contrast, male participants exhibited significant changes only in Δ_TA_, with Δ_TA_ PE = 0.257 ± 0.138 and Δ_TA_ CTR = −0.144 ± 0.1194.

**Conclusion:**

These findings suggest that psychoeducational interventions have differential gender‐specific effects, underscoring the importance of personalized approaches in the treatment of BD.

## Graphical abstract

The current study evaluates the importance of a psychoeducational intervention on telomerase activity in bipolar patients. Our results show that participation in the program leads to an increase in telomerase activity, mainly in female participants, suggesting that psychoeducation may offer a promising approach to mitigate cellular aging and manage stress‐related biomarkers in bipolar disorder.

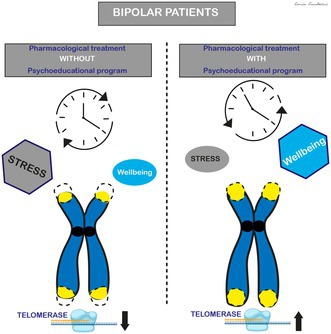

## 1 Introduction

Bipolar disorder (BD) is a severe and chronic mental illness with a lifetime prevalence of 1%–4% in the global population [1]. It is characterized by episodic changes in mood states, energy levels, and cognitive abilities [2]. Typically, with each subsequent mood episode, patients often experience increased symptom severity and an increased risk of recurrence [3]. Without appropriate treatment, individuals with BD are prone to multiple relapses, ultimately developing a range of highly disabling deficits that significantly impair their overall functioning [4, 5]. Additionally, BD is frequently accompanied by high rates of comorbidities with other conditions [6, 7].

Research suggests that BD is frequently associated with a higher prevalence of systemic disorders, commonly linked with aging, such as coronary artery disease, hypertension, metabolic imbalance, and diabetes, substantially raising the mortality risk for BD patients [8, 9, 10, 11]. While these conditions may be exacerbated by maladaptive lifestyles and/or socioeconomic adversities, which are frequently observed among BD patients [12, 13], compelling experimental evidence points to increased cellular senescence as a potential biological mechanism driving accelerated aging in this population [14, 15, 16, 17]. Supporting the hypothesis of a direct link between BD and premature aging, it has been proposed that a complex interaction between multiple biological systems and environmental triggers contributes to accelerate aging in these patients [18, 19]. Multiple studies suggest that the rate of cellular aging in BD patients is influenced by numerous environmental factors, such as chronic stress [20], lifestyle and habits [21, 22], childhood trauma, substance use, and prenatal factors [23, 24].

Particularly relevant is the link between BD and chronic stress, which has been widely associated with telomere shortening [20]. Due to their progressive shortening over time, telomeres are consistently regarded as reliable biomarkers of aging in humans [25]. Interestingly, numerous studies have established a strong association between BD and telomere shortening [26, 27, 28], reinforcing the connection with accelerated cellular aging. This suggests that chronic stress plays a dual role, both increasing the risk of BD and accelerating cellular aging through different mechanisms, like telomere shortening [26].

Given the critical role of stress in BD and its impact on cellular aging, interventions aimed at reducing stress levels could potentially slow these processes. In this context, the PsychoEducation (PE) program developed by Colom and Vieta in Barcelona in the early 2000s [29], demonstrated notable results in improving treatment adherence and managing stress in BD patients, with a reduction in relapse rates [30, 31].

In particular, this program focuses on enhancing the understanding of BD among patients and their families by educating participants about the disorder's nature, symptoms, triggers, and treatment options, fostering better self‐management and adherence to treatment. The implementation of the PE program demonstrated notable results in improving treatment adherence and stress management among BD patients [30, 31]. A randomized clinical trial assessing the efficacy of group PE for stabilized BD patients found significant long‐term benefits. Over a five‐year period, participants in the PE group experienced lower relapse rates, improved overall functioning, and enhanced insight into their condition compared to the control group [32].

Additionally, our previous studies also indicate that the PE program reduces stress in patients with BD and slows cellular aging processes, likely by normalizing hypothalamic–pituitary–adrenal (HPA) axis reactivity [33, 34]. This is evidenced by a normalized cortisol awakening response (CAR) profile in patients who completed the PE program compared to those treated with standard medications alone [33].

Together, these results underscore the effectiveness of PE in promoting better long‐term outcomes and recognizing early warning signs of mood episodes, ultimately contributing to improved quality of life for individuals with BD.

Building on the link between stress and accelerated aging in BD, another important area of investigation focuses on telomerase activity (TA) and its relationship with psychological distress caused by mental disorders. Telomerase, the enzyme that counters telomere shortening by adding the necessary telomeric DNA (TTAGGG repeats) to the ends of chromosomes, plays a crucial role in maintaining healthy cellular and immune functions [35]. In humans, TA is known to decrease under chronic stress [36, 37], but interestingly, it can increase following lifestyle interventions or physical exercise, as shown in several clinical and preclinical studies [38, 39, 40].

In harnessing its sensitivity to biological stress mediators, telomerase emerges as a promising target for innovative interventions aimed at preventing, alleviating, or even reversing the detrimental effects of increased cell‐senescence. Given the established correlation between stress, mental disorders, and TA, this study hypothesizes that the PE program, by enhancing stress management in patients, potentially results in changes in TA.

## 2 Materials and Methods

### 2.1 Participants and Study Design

The 62 participants were consecutively recruited from the outpatient service of the “Policlinico Umberto I” University Hospital of Rome, where the research and ethics committee approved the study (Prot. 0578/2023, Rif. 7218).

At the beginning of the study period (baseline), all patients underwent a semi‐structured anamnestic interview and an in‐depth clinical examination for the evaluation of somatic and psychopathological variables. All participants met the following eligibility criteria: (i) a diagnosis of BD type I or II during their lifetime, according to the Diagnostic and Statistical Manual of Mental Disorders 4th edition (DSM‐IV) criteria; (ii) stable pharmacological treatment; (iii) willingness and ability to provide written informed consent to participate. Exclusion criteria included: (i) DSM‐IV Axis I comorbidity; (ii) intellectual disability; (iii) significant concomitant neurological or organic diseases. This was a parallel 2‐group (experimental and control), prospective, randomized, controlled study.

**FIGURE 1:**
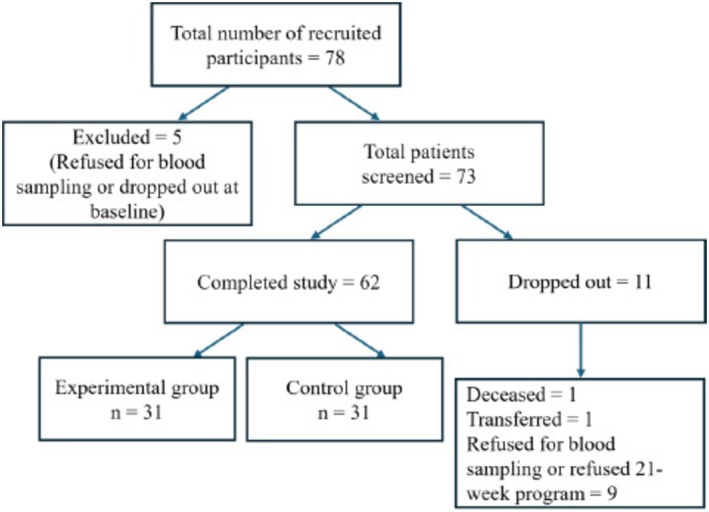
Flowchart depicting the process of recruitment, screening, and assessments of participants.

After being enrolled, both groups received standard psychiatric care and standard pharmacological treatment on a regular and stable basis, following a routine program as outpatients at the Psychiatric Clinic of the hospital, with particular regard to drug dosages, blood lithium levels, and any adverse effects, according to the main international guidelines on the treatment of BD [41] (Figure 1 and Table 1). Patients randomly assigned to the experimental group additionally received 21 sessions of PE program [29].

**TABLE 1:** Demographics and clinical characteristics of the two groups.

	Experimental group (PE) (*n* = 31)	Control group (CTR) (*n* = 31)	*p*‐value
Age	42.88 ± 11.90	47.12 ± 11.90	NS
Gender (female)	19 (61.29)	15 (48.39)	NS
Age of onset	28.88 ± 10.97	28.56 (10.92)	NS
Bipolar type (II)	4 (14.81)	9 (29.03)	NS
Duration of BD	13.57 ± 9.58	18.43 ± 12.18	NS
Family history of psychiatric disease	16 (61.53)	16 (57.14)	NS
Lithium intake	14 (45.16)	10 (32.25)	NS
Smokers	10 (32.25)	12 (38.70)	NS

*Note:* Data are presented as *n* (%) or mean ± standard deviation (SD).

Abbreviation: NS, not significant.

### 2.2 Psychoeducation Program

We implemented the well‐validated Colom and Vieta's PE program [29]. Following the original protocol, our experimental groups comprised 8 to 12 patients, engaged in 21 weekly sessions of 90 min each. Group sessions were led by experienced psychiatrists and psychologists. The PE program aimed to address four major recurring issues in BD patients: awareness of illness, adherence to treatment, early detection of prodromal symptoms and recurrences, and regularity in lifestyle. The implementation of illness components awareness focuses on helping patients understand the nature of BD, its symptoms, and its impact on their lives. This increased awareness enables patients to better recognize early signs of mood shifts and effectively manage their condition. Adherence to treatment is another crucial aspect, as non‐adherence to medication is common in BD patients. The program emphasizes the importance of consistent medication use, and it equips patients with tools to improve their commitment to their prescribed treatments, thereby reducing the risk of relapse. The early detection of prodromal symptoms and recurrences is an essential part of the program, teaching patients to identify subtle changes in their mood or behavior that may indicate the onset of a manic or depressive episode. This early detection allows for prompt intervention, potentially preventing full‐blown episodes and minimizing their impact. Finally, the program stresses the importance of regularity in lifestyle, promoting structured daily routines that include regular sleep patterns, balanced nutrition, and physical activity. Consistent habits can help stabilize mood fluctuations and reduce the risk of recurrence, acting as a protective factor against manic or depressive episodes. Throughout the 21 weeks, the PE program uses a combination of educational materials, group discussions, role‐playing exercises, and practical strategies to help patients integrate these concepts into their daily lives. The group dynamic also facilitates peer support, where patients share experiences and coping strategies, further reinforcing the learning process. By addressing these key areas, the program not only supports the management of BD but also aims to improve the overall quality of life of the patients, potentially contributing to biological changes [33, 34].

### 2.3 Isolation of Human Peripheral Blood Mononuclear Cells (PBMCs)

Venous blood samples of both the experimental group and control group patients were collected at 08.00 am. Participants were instructed to report to our laboratory after an overnight fast and to abstain from strenuous physical exercise and alcohol consumption for 24 h prior to study assessments. Subjects assigned to the experimental group reported to our research laboratory on two occasions: before the start of the first PE session (baseline—Week 1) and at the conclusion of treatment (endpoint—Week 21). Blood samples were collected from subjects assigned to the control group at the same time intervals. Blood was collected in BD Vacutainer Citrate Tubes and processed as described elsewhere (Ludlow et al., 2014). First, 5 mL of blood was transferred into a tube containing ethylenediaminetetraacetic acid (EDTA) as an anticoagulant and then diluted by adding 30 mL of Phosphate Buffered Saline (PBS) 1X (Sigma Aldrich, Milan, Italy). Subsequently, peripheral blood mononuclear cells (PBMCs) were isolated by Lymphoprep density‐gradient centrifugation (Axis‐Shield PoC AS, Oslo, Norway). Briefly, the diluted blood was gently layered over approximately 10 mL of Lymphoprep (Axis‐Shield PoC AS, Oslo, Norway) and centrifuged at 460 g for 30 min at 20°C to isolate the lympho‐monocyte ring (buffy coat) from the rest of the peripheral blood components. PBMCs were then collected using a Pasteur pipette. The harvested cell fraction was diluted with PBS 1X and centrifuged again at 300 g for 10 min at 20°C. Cells were washed again in PBS 1X, centrifuged at 300 g for 10 min at 20°C, and the final pellet was stored at −80°C prior to use.

### 2.4 Telomerase Activity Measurement

Quantitative measurement of TA was performed using the TRAPeze Telomerase Detection Kit (Sigma‐Aldrich, Milan, Italy) based on the Telomeric Repeat Amplification Protocol (TRAP) assay, according to the manufacturer's instructions. Briefly, PBMC pellets were thawed and diluted in 200 μL of CHAPS lysis buffer 1X available with the kit (containing Tris–Hcl, MgCl2, Benzamidine, β‐mercaptoethanol, CHAPS, glycerol) for every million cells. After 30 min on ice, samples were centrifuged at 12000 g for 20 min at 4°C. The supernatant (containing the extracted telomerase) was then transferred to another tube. An equal amount (2 μL) of sample was used for each patient. The products of the enzymatic activity were amplified by PCR. The used amplification protocol was 30°C for 30 min, 95°C for 2 min followed by 45 cycles at 94°C for 15 s and finally 45 cycles at 56°C for 30 s and 72°C for 1 min. PCR products were stored at 4°C until loading on 12.5% polyacrylamide gels (homemade: Resolving Gel consisting of 8.4 mL H_2_O, 10.8 mL Acrylamide, 4.8 mL TBE 5X, 800 μL APS, 30 μL TEMED; Stacking Gel consisting of 2.2 mL H_2_O, 2.2 mL Acrylamide, 1.2 mL TBE 5X, 200 μL APS, 6 μL TEMED). At the end of the electrophoresis, gels were stained with BrdU (1 μg/mL) for 1 h at RT, washed in distilled water (ddH_2_O) and then visualized using iBright Analysis Software (Thermo Fischer Scientific, Milan, Italy). The relative TA of cell lysates was quantified by Image Studio Lite software 5.0 (LI‐COR Biosciences), measuring the telomerase ladder bands signal normalized to a 36‐bp internal TRAP standard. Hela cell extracts were used as telomerase‐positive controls [42].

### 2.5 Statistical Analysis

Statistical analyses were performed with GraphPad Prism 8.0 (GraphPad Software Inc., San Diego, CA). The normality of the data was assessed using the Shapiro–Wilk test. Depending on the normality of the data, parametric tests (one‐way ANOVA for continuous variables and Chi‐square test for dichotomous variables) or non‐parametric tests (Kruskal‐Wallis test for continuous variables and Fisher's exact test for dichotomous variables) were applied. Differences were considered significant at a *p*‐value of < 0.05. Data are expressed as means ± standard deviations (m ± SD) for continuous variables and as numbers and percentages—that is, *n* (%) for dichotomous variables.

## 3 Results

A total of 62 BD patients completed the 21‐week study period. A comparison of the demographic profiles showed that participants in both the experimental (PE) and control (CTR) groups were approximately matched for all investigated variables such as age, gender, age of onset of mental illness, duration of illness, and history of psychiatric diseases. Moreover, there were no statistically significant differences between the two groups for variables that could influence telomerase activity, such as lithium intake and smoking status (Table 1).

**FIGURE 2:**
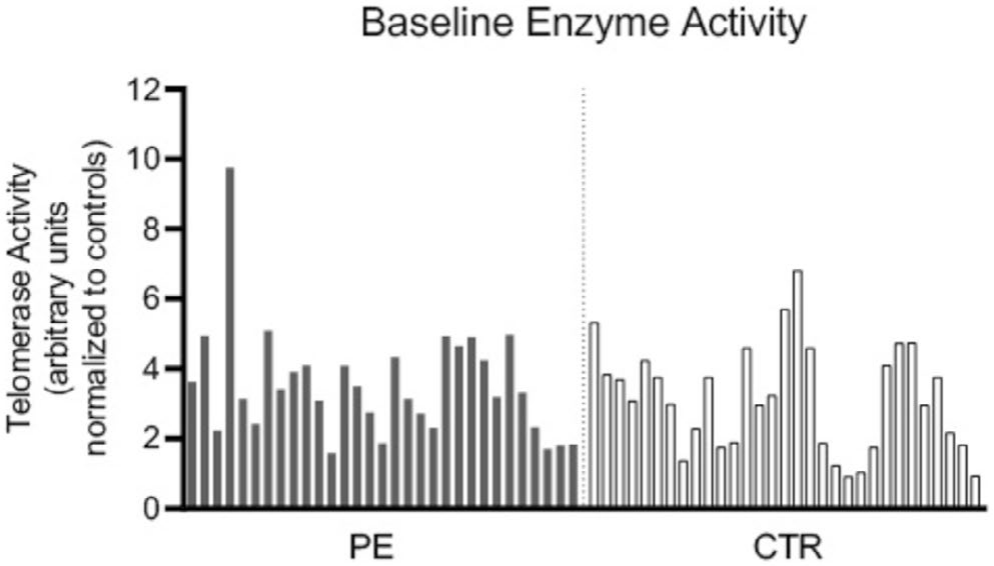
Bar graph of telomerase enzyme activity in PE and CTR groups at baseline (pre‐treatment).

As shown in Figure 2, the quantitative analysis of TA at enrollment (baseline—Week 1) was highly heterogeneous across all patients. As a result, no significant differences were found between the PE group and the CTR group at baseline for TA (TA PE = 3.546 ± 0.285; TA CTR = 3.155 ± 0.274, *p* = 0.3269).

**FIGURE 3:**
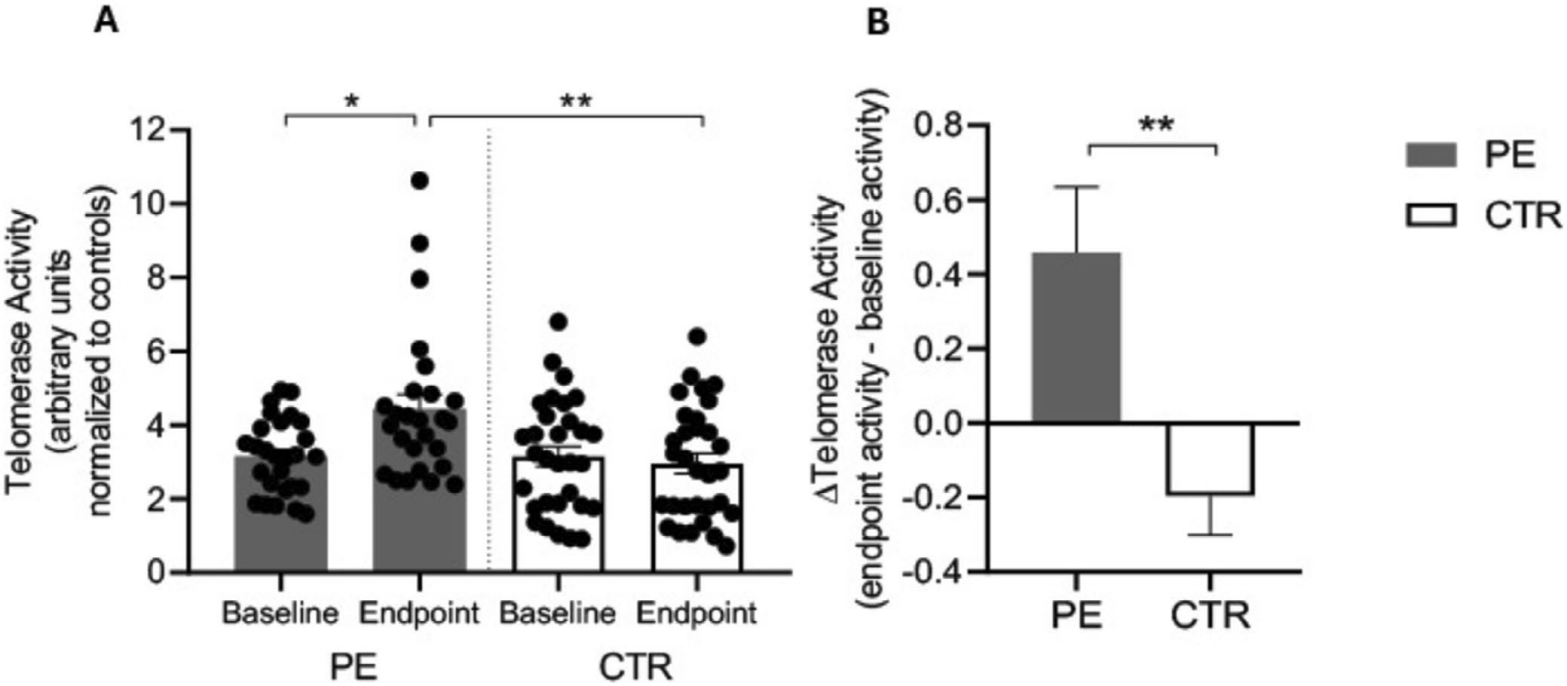
(A) Histograms represent the quantification of TA (mean ± SD) in PE (gray bars) and CTR (white bars) groups both at baseline (pre‐treatment) and endpoint (post‐treatment). Significance is shown as a *p* value calculated using a one‐way ANOVA test: *p < 0.05; **p < 0.01. (B) Histograms represent the Δ_TA_ calculated as the difference between endpoint and baseline enzyme activity in PE (gray bar) and CTR (white bar) groups. Significance is shown as a *p*‐value calculated using an unpaired t test: **p < 0.01.

However, at the end of the study (endpoint—Week 21), TA was significantly higher in patients who completed the PE program compared to the CTR group (TA PE = 4.432 ± 0.399; TA CTR = 2.959 ± 0.275, *p* = 0.0031) (Figure 3A). To further investigate these changes, we calculated the Δ_TA_ for each participant as the difference between endpoint and baseline TA, as shown in Figure 3B. Our results indicate that patients in the PE group experienced an increase in TA following the completion of the PE program, while participants in the CTR group exhibited the opposite trend, with a decreased TA (Δ_TA_ PE = 0.458 ± 0.176; Δ_TA_ CTR = −0.196 ± 0.104, *p* = 0.0022).

**FIGURE 4:**
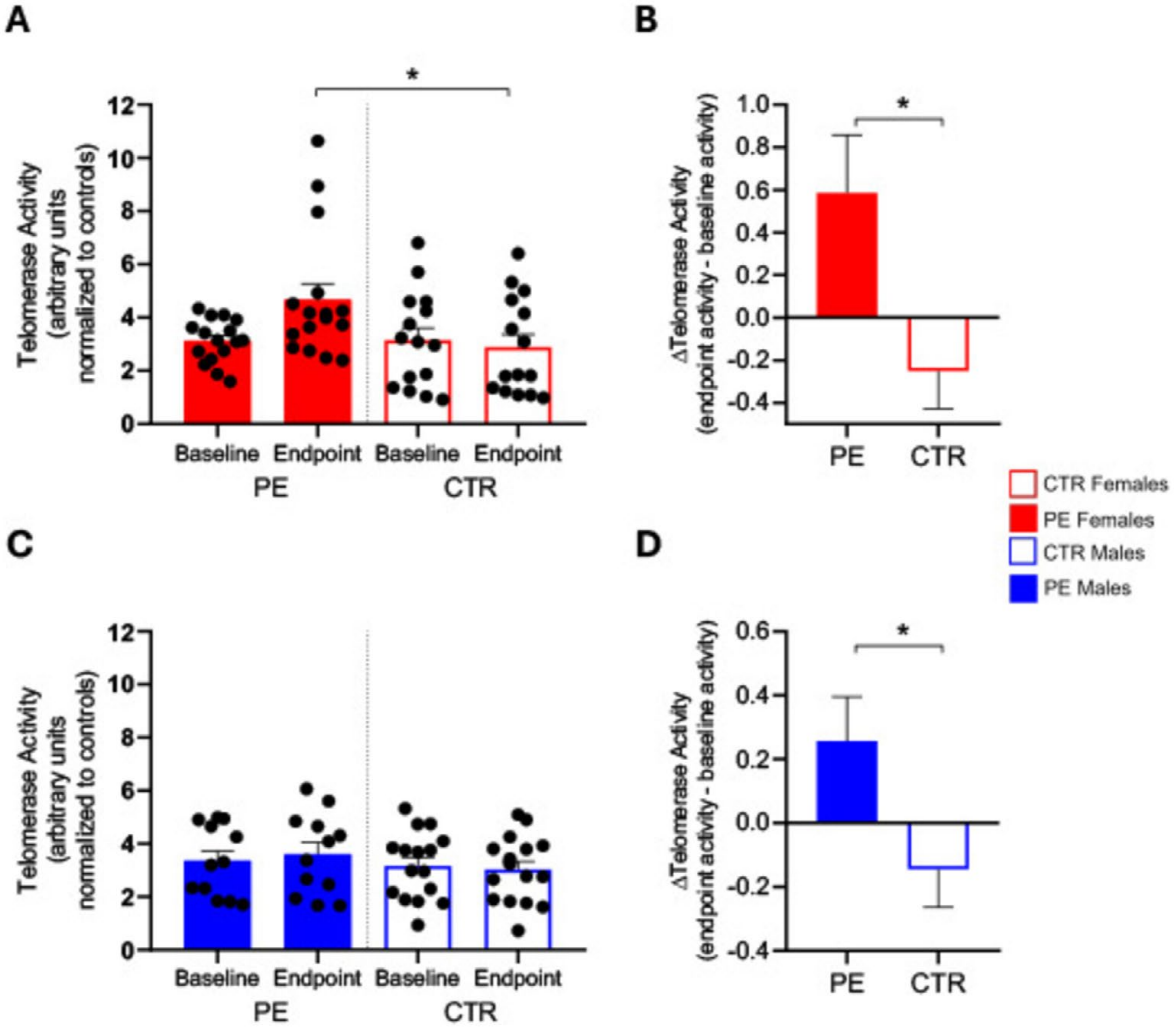
(A) Histograms represent the quantification of TA (mean ± SD) in females PE (red bars) and females CTR (red and white bars) both at baseline (pre‐treatment) and endpoint (post‐treatment). Significance is shown as a *p*‐value calculated using a one‐way ANOVA test: *p < 0.05. (B) Histograms represent the Δ_TA_ calculated as the difference between endpoint and baseline enzyme activity in females PE (red bar) and females CTR (red and white bar). Significance is shown as a *p* value calculated using an unpaired t test: *p < 0.05. (C) Histograms represent the quantification of TA (mean ± SEM) in males PE (blue bars) and males CTR (blue and white bars) both at baseline (pre‐treatment) and endpoint (post‐treatment). (D) Histograms represent the Δ_TA_ calculated as the difference between endpoint and baseline enzyme activity in males PE (blue bar) and males CTR (blue and white bar). Significance is shown as a *p*‐value calculated using an unpaired *t*‐test: *p < 0.05.

In separate analyses for males and females, we found a significantly increased TA at the endpoint in females of the PE group compared to females of the dCTR group (TA females PE = 4.668 ± 0.600; TA females CTR = 2.889 ± 0.471; *p* = 0.0392) (Figure 4A), as well as for the Δ_TA_ difference between endpoint and baseline enzyme activity (Δ_TA_ females PE = 0.586 ± 0.273; Δ_TA_ females CTR = −0.251 ± 0.177, *p* = 0.0214) (Figure 4B). Conversely, for males, we found no significant differences in the TA activity at the endpoint between the PE and CTR groups (TA males PE = 3.608 ± 0.443; TA males CTR = 3.024 ± 0.3125; *p* = 0.6988) (Figure 4C) but only for Δ_TA_ (Δ_TA_ males PE = 0.257 ± 0.138; Δ_TA_ males CTR = −0.144 ± 0.1194, *p* = 0.0373) (Figure 4D).

## 4 Discussion

The present research revealed a significant increase in TA among BD patients who participated in the PE program compared to those in the control group. These findings support the hypothesis that PE interventions aimed at reducing stress [29, 30] can positively affect biomarkers of cellular aging, such as telomerase activation [37, 38, 39, 40]. The observed increase in TA aligns with previous studies that connect stress reduction to enhanced TA [43, 44].

Chronic stress has long been associated with accelerated cellular aging, primarily through its impact on telomere shortening and reduced TA [26, 37]. In BD patients, elevated stress levels often result from the recurrent nature of mood episodes and their associated functional impairments [2, 4]. Our results suggest that the PE program not only helps in managing these stressors but also mitigates some biological effects of stress, particularly on TA. This is consistent with our recent studies showing that the PE program reduces vulnerability to stress in BD, normalizing their CAR and consequently the HPA axis reactivity [33, 34].

The positive effect of the PE program on TA could be partly explained by the program's focus on stress management and lifestyle regularity—factors known to influence TA. Previous studies have demonstrated that lifestyle interventions, such as increased physical activity and mindfulness practices, can enhance telomerase enzyme activation [44, 45, 46]. It is likely that the PE program utilized in our study has produced similar effects, helping patients to better manage stress and maintain consistent routines—critical elements in reducing the impact of BD on cellular health [46].

The influence of PE on cellular aging may also relate to its ability to decrease perceived stress, a key factor associated with telomere shortening and reduced TA [37]. Colom and Vieta's psychoeducational model emphasizes early detection of mood episodes, treatment adherence, and stress management, all of which can alleviate the psychological and physiological burden of BD [32]. This is in line with our findings, which show an increase in TA in the PE group, while the control group exhibited a decrease, likely due to ongoing exposure to unmanaged stressors.

Telomerase plays a crucial role in maintaining telomere length, an important indicator of cellular aging. Reduced TA has been linked to age‐related diseases and has been observed in patients with mood disorders [26]. The observed increase in TA may represent a strategy to counteract the accelerated cellular aging typical of these patients. While further research is needed to clarify the precise mechanisms by which stress management influences TA, our results suggest that PE interventions could represent a promising tool for mitigating some biological effects of BD, particularly those related to cellular aging. Moreover, the observed opposite trend in the CTR group, with a decrease in TA, could reflect the natural course of BD in the absence of a target intervention, such as group PE. Chronic mood instability and stress, common features in BD, may contribute to telomere attrition. In line with this, Kose Cinar (2018) found shorter telomeres in patients with late‐stage BD compared to early‐stage patients, suggesting that recurrent mood episodes accelerate cellular aging [45]. Future studies should investigate the effects of PE on telomere length, as well as its impact on other markers of cellular aging in BD patients.

The results from this study highlight significant gender differences in TA responses to the PE program in BD patients. Notably, females exhibited a significant increase in TA post‐intervention compared to their control counterparts, indicating that the psychoeducational approach may have a particularly beneficial impact on female BD patients. The calculated Δ_TA_ for females was PE = 0.586 ± 0.273; CTR = −0.251 ± 0.177, demonstrating a marked enhancement in TA in the PE group. This finding aligns with previous research suggesting that gender may influence the biological responses to stress and psychological interventions [46, 47]. In contrast, while there were no significant differences in TA at the endpoint between the PE and CTR groups for males, we did find significant changes in Δ_TA_, with a value of PE = 0.257 ± 0.138; CTR = −0.144 ± 0.1194, suggesting that the PE program had a measurable effect on the change in TA over time for males. These gender‐specific effects raise relevant questions regarding the underlying biological and psychosocial mechanisms. From a biological perspective, one potential explanation for the gender differences in TA is the influence of sex hormones. Estrogens have been linked to TA and are known to promote telomere maintenance, which may contribute to the more significant increases in TA observed in females [48]. Typically, higher estrogen levels in females could represent a key factor potentially explaining their greater response to PE in terms of TA observed in our results. However, while these hormonal influences are well‐documented in other contexts, further research is needed to confirm whether estrogen directly impacts TA in patients with BD. Moreover, research into the pathophysiology of BD has highlighted gender differences in brain structure and function, which could also affect how each gender responds to psychoeducational interventions. For example, Lee et al. (2024) found that males BD patients exhibited a larger grey matter volume and an altered functional connectivity in the limbic system compared to females [49]. These brain region differences could influence the biological mechanisms underlying the observed gender differences in TA response to PE. However, our study did not include neuroimaging data, and future studies could investigate whether these structural differences in the brain correlate with TA changes in both genders. Psychosocial factors might also play a role in the observed gender differences in TA. For instance, it is well known that gender affects each element in the stress process, even influencing coping responses and the health implications of stress reactions [50, 51]. Numerous studies have reported many differences in how women and men deal with stress; for example, women have been found to be more active and problem‐focused than men in their coping strategies [52]. This may lead to women better responding to therapies focusing on managing psychological stress, such as our group psychoeducational program, possibly reflecting on the observed increase in TA. In addition, it is known that both telomere length and TA could be influenced by the lithium treatment [53, 54]. However, in our study, there were no differences in the percentages of lithium intake between the PE and CTR groups, strengthening the efficacy of the PE program in influencing TA.

Despite our interesting findings, several limitations should be acknowledged. Firstly, the sample size is relatively small, and the study was conducted at a single site, which may limit the generalizability of the results. This sample size is consistent with similar studies in the field, but replication in larger, multi‐center studies is necessary to validate these findings. Additionally, the 21‐week duration of the study is relatively short for evaluating the sustained impact of PE program on TA. A long‐term follow‐up would provide more insights into the intervention's lasting effectiveness.

While significant changes in TA were observed, the exact mechanisms by which PE influences telomerase activation remain unclear. Longitudinal studies examining a broader range of biomarkers may help elucidate the underlying pathways involved. Another limitation of our study is the absence of psychological and cognitive evaluations conducted before and after the PE intervention.

Since the effect of PE on TA can be mediated by changes in cognitive performance, as well as by improvements in depressive and manic symptoms, reduction of relapses, and increased adherence to pharmacological treatment, further studies will need to analyze any changes in these factors before and after PE. However, despite the lack of psychological measures performed before and after the implementation of PE program, possible confounding variables such as adherence to treatment, improvement of symptoms, and mood changes were controlled throughout the duration of the study, as far as possible, through routine meetings carried out on a regular basis with patients in both groups.

The potential relationship between stress, TA, and PE warrants further exploration for improved clarity and understanding. For instance, future studies could explore whether different components of the PE program (such as stress management, lifestyle regularity, or treatment adherence) have varying effects on TA, providing a more detailed understanding of how PE influences cellular aging in patients with BD.

## 5 Conclusions

In conclusion, this study provides compelling evidence that participation in a PE program can significantly enhance TA in individuals with BD, indicating a potential avenue to mitigate cellular aging in this population. The observed gender differences, with females exhibiting significant changes in both pre‐and post‐intervention TA and Δ_TA_, highlight the importance of tailoring interventions to accommodate these variations. Understanding these gender‐specific responses is crucial for developing effective strategies to enhance the well‐being and long‐term outcomes of patients with BD. Future research should further explore these dynamics, particularly focusing on the underlying mechanisms driving these differences in TA in response to stress and PE interventions.

## Conflicts of Interest

The authors declare no conflicts of interest.

## Data Availability

The data that support the findings of this study are available on request from the corresponding author. The data are not publicly available due to privacy or ethical restrictions.
